# Theta-specific susceptibility in a model of adaptive synaptic plasticity

**DOI:** 10.3389/fncom.2013.00170

**Published:** 2013-11-21

**Authors:** Christian Albers, Joscha T. Schmiedt, Klaus R. Pawelzik

**Affiliations:** ^1^Department of Neurophysics, Institute for Theoretical Physics, University of BremenBremen, Germany; ^2^Schmid lab, Ernst Strüngmann Institute for Neuroscience in Cooperation with Max Planck SocietyFrankfurt am Main, Germany

**Keywords:** synaptic plasticity, STDP, learning, memory, theta oscillation

## Abstract

Learning and memory formation are processes which are still not fully understood. It is widely believed that synaptic plasticity is the most important neural substrate for both. However, it has been observed that large-scale theta band oscillations in the mammalian brain are beneficial for learning, and it is not clear if and how this is linked to synaptic plasticity. Also, the underlying dynamics of synaptic plasticity itself have not been completely uncovered yet, especially for non-linear interactions between multiple spikes. Here, we present a new and simple dynamical model of synaptic plasticity. It incorporates novel contributions to synaptic plasticity including adaptation processes. We test its ability to reproduce non-linear effects on four different data sets of complex spike patterns, and show that the model can be tuned to reproduce the observed synaptic changes in great detail. When subjected to periodically varying firing rates, already linear pair based spike timing dependent plasticity (STDP) predicts a specific susceptibility of synaptic plasticity to pre- and postsynaptic firing rate oscillations in the theta-band. Our model retains this band-pass property, while for high firing rates in the non-linear regime it modifies the specific phase relation required for depression and potentiation. For realistic parameters, maximal synaptic potentiation occurs when the postsynaptic is trailing the presynaptic activity slightly. Anti-phase oscillations tend to depress it. Our results are well in line with experimental findings, providing a straightforward and mechanistic explanation for the importance of theta oscillations for learning.

## 1. Introduction

Synaptic plasticity likely is the key neural substrate underlying learning and memory in the brain. Early ideas on the problem of synaptic plasticity posited that positive correlations between neuronal activities are the signal for the synapse to potentiate (see e.g., review by Markram et al., 2011); later experiments showed that the relevant signal is not just the average correlation of activity, but rather the precise temporal order of single spikes at the pre- and postsynaptic neuron (Markram et al., 1997; Bi and Poo, 1998; Zhang et al., 1998; Feldman, 2000). This phenomenon was termed Spike Timing Dependent Plasticity (STDP) and subsumed in the well-known exponential spike pair STDP window [Song et al. (2000), spSTDP in the following]. In many theoretical studies, this window serves as a look-up table to compute the weight change: Identify any pair of a pre- and a postsynaptic spike, locate the time difference between the two spikes in the STDP window and add up the respective weight changes [see Morrison et al. (2008) for a review of implementations]. While this linear approach has its appeal, it is not sufficient, because the contributions of spikes in sequences do not simply add up (Wang et al., 2005). Some experiments find that a spike can suppress the effect of later spikes of the same synaptic side (Froemke and Dan, 2002; Froemke et al., 2006). Other experiments show that contrary to expectation a single pre-post pair fails to potentiate the synapse, but a pre-post-post triplet leads to strong long term potentiation (LTP) (Sjöström et al., 2001; Nevian and Sakmann, 2006; Wittenberg and Wang, 2006). These findings highlight the need for any accurate model of STDP to include non-linearities. There are several different models available which attempt to capture the experimental results. One class of models contains phenomenologically motivated non-linear extensions of spSTDP which are tailored to explain experimental data (Froemke et al., 2006; Pfister and Gerstner, 2006; Schmiedt et al., 2010). A second class are calcium-based models, which are grounded on biophysical considerations. Most of these models invoke the calcium control hypothesis, which states that a moderate increase of the calcium concentration in the postsynaptic spine leads to long term depression (LTD), while high concentrations lead to LTP. The models then are concerned with the details of the calcium dynamics (Shouval et al., 2002; Cai et al., 2007; Graupner and Brunel, 2012; Uramoto and Torikai, 2013). A third class of models includes neuronal signals beyond spikes, most prominently the postsynaptic membrane potential (Clopath et al., 2010). There are few experimental studies which quantitatively examine the synaptic change in response to complex and versatile spike patterns (Froemke and Dan, 2002; Wang et al., 2005; Froemke et al., 2006; Nevian and Sakmann, 2006), however, none of the models covers all data sets [for the model of Uramoto and Torikai (2013), see Discussion]. An attenuated synaptic response to repeated high frequency spiking [Short term depression, (Tsodyks and Markram, 1997; Tsodyks et al., 1998; Zucker and Regehr, 2002)] is explicitly included in several models (Froemke et al., 2006; Cai et al., 2007; Schmiedt et al., 2010), which however, do not explain the full range of experiments. In the following, we present a minimal dynamical model which includes pre- and postsynaptic adaptation as well as an activating contribution, hence we call it contribution dynamics model (CD model). Some of the elements of this model can be found in previous work (Schmiedt et al., 2010). We evaluate the validity of the model by fitting it to the four different data sets mentioned above, and compare its performance with the Triplet model of Pfister and Gerstner (2006), which is similar in scope and formulation, but lacks adaptation.

Another open question in neuroscience addresses the neural substrate for the known importance of oscillatory brain states for memory formation (Fell and Axmacher, 2011; Colgin, 2013). Many studies find that the mere presence of oscillations of increased theta power is enough to enhance the learning process, even if the oscillations are present *before* (and during) the learning trial (Seager et al., 2002; Nokia et al., 2008; Guderian et al., 2009). Other studies find that not theta power, but global theta synchronization promote good learning efficacy (Mölle et al., 2002; Burke et al., 2013). It is likely that theta synchronization is imposed on the affected brain areas by some higher area, which causes the synchronization with a phase difference of around zero, such that maxima of activity in synchronized areas occur at the same time (Fell and Axmacher, 2011). It was suggested that the reason is a specific phase dependence of synaptic plasticity in theta oscillations: If activity maxima in the pre- and postsynaptic neurons co-occur, the synapse potentiates, if the presynaptic neuron bursts during the trough of the theta oscillation, the synapse depresses (Pavlides et al., 1988; Hyman et al., 2003).

Can the combination of these findings be explained by a single mechanism? We address this question with the hypothesis that the reason lies in the filter properties of synaptic plasticity, which can be investigated with models of synaptic plasticity. To test this we assume that theta-band oscillations in large scale signals like EEG or ECoG are caused by corresponding periodic modulation of neuronal activity. For simplicity we neglect spike-spike correlations and assume stochastic spiking. We investigate the synaptic susceptibility to oscillations from the delta band to the gamma band (1–80 Hz) in spSTDP and in the CD model. For comparison, we did the same with a range of other models (Shouval et al., 2002; Pfister and Gerstner, 2006; Cai et al., 2007; Graupner and Brunel, 2012). We found that for spSTDP with physiological parameters synapses are susceptible to oscillations in the theta band (4–8 Hz). The same susceptibility is evident also in the CD model, which however, shifts the phase dependence of LTP close to zero phase difference, in accordance with experimental results. By removing single contributions from the CD model and investigating the resulting changes of the susceptibility, we find that presynaptic adaptation and a conditional activation are the necessary prerequisites for phase zero susceptibility.

## 2. Materials and methods

In the following, we use a short hand notation to denote spike patterns. “Pre” or “Post” refer to the origin of the spike, the pre- or postsynaptic neuron. A string like “pre-post” denotes first a presynaptic spike a postsynaptic spike, regardless of exact timing. “Post-pre-post-post” describes a postsynaptic spike, then a presynaptic spike, followed by two postsynaptic spikes.

### 2.1. Modeling spike pair STDP with differential Hebbian learning

The differential Hebbian learning rule is a rather simple algorithm for weight changes (Kosko, 1986). The synapse changes proportional to the product of the presynaptic activity and the temporal derivative of the postsynaptic activity. For spiking neurons, however, this makes little sense, and one has to introduce some kind of low pass filtering of neuronal activities to gain a signal suitable to calculate synaptic change. As usual, we use delta pulses to model neuronal spike trains:

(1) $$x_{i}(t)=\sum_{k}\delta (t-t_{i}^{k}),$$

where *i* ∈ {pre, post} denotes the location of the spiking event. Each spikes leaves an exponential trace *y*_*i*_ on its synaptic side, which can be described by the differential equation

(2) $${\dot{y}}_{i}=-\frac{y_{i}}{\tau_{i}}+x_{i}.$$

We use the dot notation to denote temporal derivatives. The weight change is given by

(3) $$\dot{w}\propto y_{\text{pre}}\cdot {\dot{y}}_{\text{post}}.$$

This simple system of equations is equal to (balanced) spSTDP, as we show now. Consider the solution of Equation (2) to a single spike at time *t*_*i*_:

(4) $$y_{i}=\Theta (t-t_{i})e^{-\frac{t-t_{i}}{\tau_{i}}}.$$

Here, Θ(*t*) is the Heaviside function, i.e., Θ(*t*) = 0 for *t* < 0 and Θ(*t*) = 1 everywhere else. The weight change is calculated via

(5) $$\Delta w=c_{w}\int_{-\infty }^{+\infty }y_{\text{pre}}{\dot{y}}_{\text{post}}dt,$$

where we introduce the constant of proportionality *c*_*w*_. The weight change resulting from a pair of one pre- and one postsynaptic spike is given by

(6) $$\Delta w=c_{w}\{\begin{array}{ll} \left(1-\frac{1}{1+\frac{\tau_{\text{post}}}{\tau_{\text{pre}}}}\right)\exp\left(-\frac{t_{\text{post}}-t_{\text{pre}}}{\tau_{\text{pre}}}\right) & \text{for }t_{\text{pre}}<t_{\text{post}}\\ -\frac{1}{1+\frac{\tau_{\text{post}}}{\tau_{\text{pre}}}}\exp\left(-\frac{t_{\text{pre}}-t_{\text{post}}}{\tau_{\text{post}}}\right) & \text{for }t_{\text{pre}}>t_{\text{post}}. \end{array}$$

This is the standard STDP window for balanced spSTDP, where the areas under the LTP and LTD part of the curve are of exactly equal size, and the decay time constants are given by τ_pre_ and τ_post_ for LTP and LTD, respectively. Due to the linearity of the equations, the learning rule is also completely linear, and every spike pair in a given spike pattern is treated the same by the learning rule.

The STDP window in differential Hebbian learning is determined by three parameters. To scale the LTD and LTP parts of the window relative to each other a fourth parameter is needed. We split the weight change into a depression and a potentiation part by inserting Equation (2) into (3) and introduce a scale parameter *q*:

(7) $$\dot{w}=c_{w}y_{\text{pre}}\left(qx_{\text{post}}-\frac{y_{\text{post}}}{\tau_{\text{post}}}\right)\text{​}.$$

This manipulation changes the STDP window to:

(8) $$\Delta w=c_{w}\{\begin{array}{ll} \left(q-\frac{1}{1+\frac{\tau_{\text{post}}}{\tau_{\text{pre}}}}\right)\exp\left(-\frac{t_{\text{post}}-t_{\text{pre}}}{\tau_{\text{pre}}}\right) & \text{for }t_{\text{pre}}<t_{\text{post}}\\ -\frac{1}{1+\frac{\tau_{\text{post}}}{\tau_{\text{pre}}}}\exp\left(-\frac{t_{\text{pre}}-t_{\text{post}}}{\tau_{\text{post}}}\right) & \text{for }t_{\text{pre}}>t_{\text{post}}. \end{array}$$

Adjusting *q* scales the LTP part of the STDP window as required. For example, setting *q* = 1/(1 + τ_post_/τ_pre_) cancels LTP for every possible spike pattern. There are experiments which show that for low frequencies of spike pair induction, pre-post pairs do not change the synapse, while post-pre pairs still depress the synapse (Sjöström et al., 2001; Nevian and Sakmann, 2006; Wittenberg and Wang, 2006). However, other spike patterns in these studies potentiate the synapse, which suggests that in order to generalize this description of STDP, one has to turn *q* into a function of time.

### 2.2. The contribution dynamics model

For the CD model we use the differential Hebbian learning rule described above as basis, and extend it by several new equations. First, we introduce an adaptation variable *u*_*i*_ for each synaptic side. This dynamics resemble those of the presynaptic resources in models of synaptic short term depression (Tsodyks and Markram, 1997; Tsodyks et al., 1998). Its effect is the attenuation of the impact of rapid spiking on the synapse, and we model it by

(9) $${\dot{u}}_{i}=\frac{1-u_{i}}{\tau_{i}^{\text{rec}}}-c_{i}u_{i}(t-0)x_{i}.$$

The update of the trace Equation (2) is now changed to

(10) $${\dot{y}}_{i}=-\frac{y_{i}}{\tau_{i}}+u_{i}(t-0)x_{i},$$

where we write *u*_*i*_ (*t* − 0) to emphasize that in order to update each variable in case of a spike, one has to use the value of *u*_*i*_ shortly before the spike.

If the learning rule Equation (3) was left unchanged, the effect of the adaptation variables *u*_*i*_ would be a rescaling (shrinking) of the STDP window with consecutive spikes, and relaxation during silence. This property removes the linearity of the original STDP learning rule, as the influence of each spike on the synapse depends on the history of spiking of the respective neuron. We introduce an additional non-linearity, by allowing *q* (Equation 7) to vary over time:

(11) $$\dot{q}=\frac{q_{\text{min}}-q}{\tau_{q}}+c_{q}\Theta (y_{\text{pre}}-\vartheta_{q})x_{\text{post}},$$

where Θ is again the Heaviside function. *q* is a trace of the postsynaptic activity conditional on the presynaptic trace: Only if *y*_pre_ > ϑ_*q*_ at the time of a postsynaptic spike, *q* increases. For all other times, it relaxes back to *q*_min_. The actual weight change is finally given by

(12) $$\dot{w}=c_{w}y_{\text{pre}}\left(q(t-0)u_{\text{post}}(t-0)x_{\text{post}}-\frac{y_{\text{post}}}{\tau_{\text{post}}}\right)\text{​}.$$

The specific formulation of *q* is motivated by its simplicity—linear ordinary differential equation of first order for the decay term—and several observations in the data of Nevian and Sakmann (2006). In these experiments, a pre-post pair does not change the synapse, but the pre-post-post triplet does. The translation to an STDP framework is that the LTP part of the STDP window needs to vanish when the synapse is relaxed (any previous activity took place relatively long ago), but reappear in reaction to certain activity patterns. In this example (pre-post vs. pre-post-post), the desired outcome can be achieved by Equation (11) without the Heaviside function: $\dot{q}$ = (*q*_min_ − *q*)/τ_*q*_ + *c*_*q*_*x*_post_. However, in the case of a post-post-pre-post pattern (Figure 2C third data point from left) this would lead to a huge upscaling of the LTP part, which was not observed. This prompted us to install the threshold such that recent presynaptic activity gates the increase of *q*. We chose the all-or-none threshold to exclude any non-linear effects of *q* on the STDP window. Because of the upregulation of potentiation, we call *q* the activation variable.

**Figure 1:**
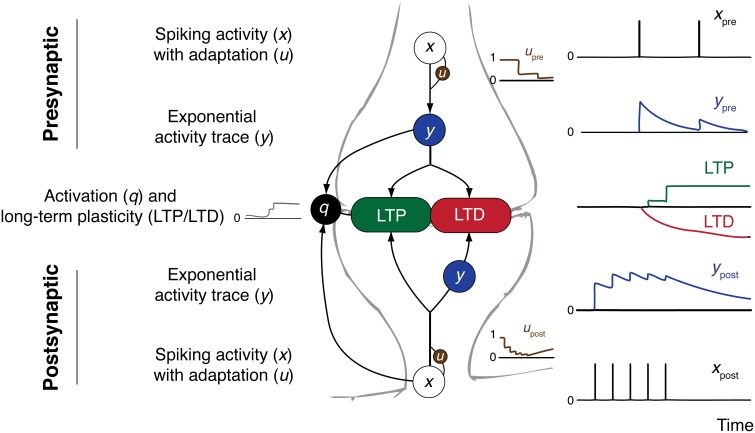
**Overview over the CD model and its constituents**. Presynaptic **(top)** and postsynaptic **(bottom)** contributions to synaptic plasticity are shown. Arrows indicate direction of influence according to model equations. On the right side example spike trains and resulting traces of the variables are shown.

Figure 1 gives an overview over the components of the CD model.

### 2.3. Fitting the CD model to experimental data

To evaluate the ability of the CD model to reproduce experimental findings, we matched its parameters to the following four *in vitro* data sets:

- Visual cortex of young rats, thick tufted cells in layer 5 [VC5, Sjöström et al. (2001)].
- Hippocampal neurons of rat embryos in culture [HC, Wang et al. (2005)].
- Somatosensory cortex of young rats, pyramidal cells in layer 2/3 [SC23, Nevian and Sakmann (2006)].
- Visual cortex of young rats, pyramidal cells in layer 2/3 [VC23, Froemke et al. (2006)].

In these experimental studies, the change of synaptic efficacy is given as the ratio of the isolated EPSP (which we assume to be proportional to the synaptic weight) after and before the induction protocol:

(13) $$\begin{array}{l} \text{EPSP ratio}=\frac{{\text{EPSP}}_{\text{after}}}{{\text{EPSP}}_{\text{before}}}=\frac{w_{\text{after}}}{w_{\text{before}}}=\frac{w_{\text{before}}+\Delta w}{w_{\text{before}}}\\ \;=1+\frac{\Delta w}{w_{\text{before}}}. \end{array}$$

We identify Δ*w* with the weight change in the CD model. Additionally, we assume that the synaptic weight before the induction has a fixed value. The goal of the fitting process is to compare the experimental synaptic change with the model prediction Δ*w^CD^* and find the set of parameters π = {τ^rec^_pre_, *c*_pre_, τ^rec^_post_, …} which minimizes the error

(14) $$\text{E}=\frac{1}{N}\sum_{i=1}^{N}{\left(\frac{\Delta w_{i}^{\text{exp}}-\Delta w_{i}^{CD}(\pi )}{{\text{SEM}}_{i}}\right)}^{\text{​}2}\text{​},$$

where *N* is the number of experiments in the data set, *i* the index of the experiment and SEM_*i*_ the published standard error of the mean for experiment *i*. The minimization of the error was done by a brute force search in the space of parameters. The bounds we defined for the search are given in Table 1 (see Appendix for additional remarks on the bounds). For each data set, some parameters were set by hand to fixed values as follows: We set *q*_min_ according to the outcome of the pre-post spike pair experiment found in every data set. If the pre-post spike pair resulted in no change of synaptic efficacy, *q*_min_ = 1/(1 + τ_post_/τ_pre_), otherwise *q*_min_ = 1. The time constants τ_pre_ and τ_post_ turn up as the decay constants of the STDP window (see Equation 8). For the experiments in VC23 and HC these values were explicitly given, so we did not change them. For the other two cortical data sets, we used the values of the experiment in VC23. As each spike pattern contained only one presynaptic spike, no information could be obtained for presynaptic adaptation for the data from SC23. This left for fitting here τ^rec^_post_, *c*_post_, τ_*q*_, *c*_*q*_, ϑ_*q*_ and *c*_*w*_. In all other data sets, τ^rec^_pre_ and *c*_pre_ were additionally fitted.

**Table 1:** **Bounds on parameters in the CD model**.

**Parameter**	**Unit**	**Min**	**Max**
τ^rec^_pre_	s	0.001	3
*c*_pre_		0	1
τ^rec^_post_	s	0.001	3
*c*_post_		0	1
τ_*q*_	s	0.001	3
*c*_*q*_		0	10
ϑ_*q*_		0	0.2
*c*_*w*_		0.001	0.1

### 2.4. Fitting the triplet model to experimental data

The CD model is structurally similar to the Triplet model conceived by Pfister and Gerstner (2006). The latter is a set of linear differential equations of first order that describe traces of activity at the synapse. Each spike leaves two traces at the synapse: *r*_1_, *r*_2_ for the presynapse and *o*_1_, *o*_2_ for the postsynapse, which interact to determine the weight change. For the update of the traces, there are two possible choices. The first one is that each spike increases its respective traces by one; this is equivalent to the *y*_*i*_ dynamics of Equation (2). Second, at the time of a spike the respective traces always jump to unity. The equation of the traces changes to *ẏ_i_* = −*y*_*i*_/τ_*i*_ + (1 − *y*_*i*_ (*t* − 0))*x*_*i*_. The first update rule is called “all to all interactions,” the second “nearest neighbor interactions.” The weight change in the Triplet model then consists of a standard spike pair STDP rule plus the spike triplet interaction, which is proportional the product *r*_1_ · *o*_2_ (*o*_1_ · *r*_2_) at the time of a postsynaptic (presynaptic) spike for LTP (LTD). The main differences between the two models are that in the CD model *y*_pre_ and *y*_post_ are subject to spike amplitude adaptation and that the triplet interactions are replaced by the (conditional postsynaptic) activation *q*. For comparison, we fitted the triplet model to the data sets VC23, SC23, and VC5. The Triplet model has been fitted to the HC data set, the parameters can be found in Pfister and Gerstner (2006). It has also already been fitted to the VC5 data set (same article). However, the spike induction protocol used for fitting was uniformly 60 spike pairs with Δ*t* = ± 10 ms delivered at different frequencies (1–40 Hz). In the study of Sjöström et al. (2001), spike pairs for frequencies greater than 1 Hz were delivered in 15 bursts of 5 pairs with varying intra burst frequency, with bursts being 10 s apart. We re-fitted the Triplet model to VC5 to better compare the two models. In contrast to the original article, we furthermore allowed the triplet interaction parameters *A*^+^_3_ and *A*^−^_3_ to become negative to account for adaptation in the data. The fitting procedure was similar to the fit of the CD model; in particular, the STDP window time constants τ_+_ and τ_−_ were not fitted, but set to predetermined values. The bounds defined for the parameters are given in Table 2.

**Table 2:** **Bounds on parameters in the Triplet model**.

**Parameter**	**Unit**	**Min**	**Max**
τ_*x*_	s	0.0001	5
τ_*y*_	s	0.0001	5
*A*^+^_2_		0	0.1
*A*^+^_3_		−0.1	0.1
*A*^−^_2_		0	0.1
*A*^−^_3_		−0.1	0.1

### 2.5. Mean weight changes in models of STDP

The formulation of spSTDP as differential Hebbian learning allows for a simple analytical treatment of continuous firing rates rather than spike events. Under the assumption of poissonian spiking and vanishing correlations between pre- and postsynaptic spikes, one can easily compute the mean of the traces *y*_*i*_:

(15) $$\langle {\dot{y}}_{i}\rangle =\langle -\frac{y_{i}}{\tau_{i}}+x_{i}\rangle =-\frac{\langle y_{i}\rangle }{\tau_{i}}+r_{i}(t),$$

where *r*_*i*_ (*t*) = 〈*x*_*i*_〉 is the continuous and time-dependent firing rate of neuron *i*. Because of the vanishing spike-spike correlations, both traces combine to give the weight change as

(16) $$\dot{w}={\dot{w}}_{+}+{\dot{w}}_{-}=c_{w}q\langle y_{\text{pre}}\rangle r_{\text{post}}-\frac{\langle y_{\text{pre}}\rangle \langle y_{\text{post}}\rangle }{\tau_{\text{post}}},$$

where 〈*y*_*i*_〉 is the solution of differential Equation (15) for a given time course of the firing rates *r*_*i*_ (*t*).

In the non-linear models of STDP [CD model, Triplet model, the three Calcium models (Shouval et al., 2002; Cai et al., 2007; Graupner and Brunel, 2012)], the numerous non-linearities in each model did not allow to compute and solve the mean field equations. We therefore computed the average weight change for a given stimulation protocol and model by generating many realizations of the same continuous and time dependent firing rates from inhomogeneous poisson processes (Dayan and Abbott, 2005), which we fed into each model. As in the analytical calculations for spSTDP, we assumed poissonian firing with vanishing spike-spike correlations from synaptic transmission. In this case the probability of finding a spike in a time bin of width Δ*t* is given by

(17) $$p(\text{spike in neuron }i\text{ in }\Delta t|t)=r_{i}(t)\Delta t,$$

where *r*_*i*_(*t*) is the firing rate of neuron *i* as a function of time.

### 2.6. STDP and theta oscillations

We hypothesize that the link between theta oscillations and learning lies in certain filter properties of the synapse, which likely depend on the model of synaptic plasticity used. We investigate synaptic filter properties in a variety of different models: spSTDP, the CD model, the Triplet model, and three different calcium models, with the aim to carve out prerequisites for a synaptic filter. We used a sinusoidal oscillation to model the firing rate. For the case of spSTDP, the firing rate is given by:

(18) $$r_{i}(t)=1+\varepsilon \cos(\omega_{\text{mod}}t-\phi_{i})\;.$$

Here, ε ∈ [0, 1] is a parameter that controls the amplitude of the oscillation, ω_mod_ = 2π*f*_mod_ is the modulation frequency, and ϕ_*i*_ is the phase of the oscillation. Because only relative phase is important for the weight change, we set ϕ_pre_ = 0 and ϕ_post_ = Δφ. We do not specify an absolute baseline firing rate for spSTDP, because it is just a scale factor and does not qualitatively change the results. The value we report is the weight change per time averaged over one period of oscillation. This is constant after transients from the onset of neuronal activity died out:

(19) $$\Delta w=\frac{1}{T}\int_{t^{\prime }}^{t^{\prime }+T}y_{\text{pre}}{\dot{y}}_{\text{post}}dt\;.$$

*T* = 1/*f*_mod_ is the period of the modulatory oscillation, and *t*' » τ_pre_, τ_post_ is chosen such that any transient behavior in the traces *y*_*i*_ due to switching on the activity are gone. We derive the analytical solution for Equation (19) in the appendix, and use it to generate the plots in Figure 4.

In the case of the non-linear models of STDP a baseline firing rate (the firing rate averaged over one period of oscillation) has to be specified. The respective firing rates of the pre- and postsynaptic neurons change to

(20) $$\begin{array}{l} r_{\text{pre}}(t)=r_{\text{base}}\left(1+\varepsilon \cos(\omega_{\text{mod}}t)\right)\\ r_{\text{post}}(t)=r_{\text{base}}\left(1+\varepsilon \cos(\omega_{\text{mod}}t)-\Delta \varphi \right). \end{array}$$

To simplify the analysis, both neurons had the same baseline firing rate and the same modulation frequency. Similar to spSTDP, in the CD model and the Triplet model we calculated the weight change per time by averaging over an integer multiple of the oscillation period starting after enough time has passed to settle the transient:

(21) $$\Delta w=\frac{1}{NT}\langle \int_{t^{\prime }}^{t^{\prime }+NT}{\dot{w}}_{\text{model}}\;dt\rangle ,$$

where *model* ∈ {*CD*, Triplet} refers to the model used. In all simulations, *t*' = 2 s and *NT* = 98 s; we simulated only integer values of the modulation frequencies, so *NT* is always a multiple of the period.

For the three calcium-based models the procedure was different. All models have inherent weight limits. As a consequence, the rate of weight change itself is a function of time which does not settle into an equilibrium other than saturation. Therefore it is not feasible to calculate an average weight change rate as with the spike pair models. We rather let the two neurons fire with periodic firing rates for some time [2 s and 5 s in the model of Shouval et al. (2002), 10 s in the model of Cai et al. (2007), 5 s in the model of Graupner and Brunel (2012)], after which we silenced the neurons, but continued to simulate until the synapse settled into an equilibrium. With all models, we report the final weight, with *w* = 1 being the initial weight.

The fitting procedure and numerical simulations (Monte-Carlo-Simulations) were done with custom-made programs in Matlab (Mathworks Inc., Natick, MA, USA). Numerical integration of non-linear differential equations was done with Eulers method and a step size of 0.1 ms. Linear differential equations were solved analytically, and time evolution of the variables was calculated based on the spike times.

## 3. Results

### 3.1. The CD model captures a wide range of dynamical phenomena

The CD model describes the non-linear interactions between spikes on either synaptic side acting on the contributions to synaptic changes. To evaluate its ability to capture synaptic changes, we chose four studies from the literature which measure synaptic changes in response to complex spike patterns, and matched the model parameters to the experimental results. Because the experiments were conducted under different conditions (brain region, presynaptic stimulation method), the parameters had to be fitted separately for each data set. In the following we describe the experiments and how the CD model recreates them in relative detail, to illustrate the action of the different contributions.

In all experimental studies, spikes were artificially induced by application of current pulses to patched neurons, or sometimes in the case of presynaptic spikes by stimulating the tissue close to the dendritic tree of the postsynaptic neuron.

#### 3.1.1. Area VC5 (Sjöström et al., 2001)

**Figure 2:**
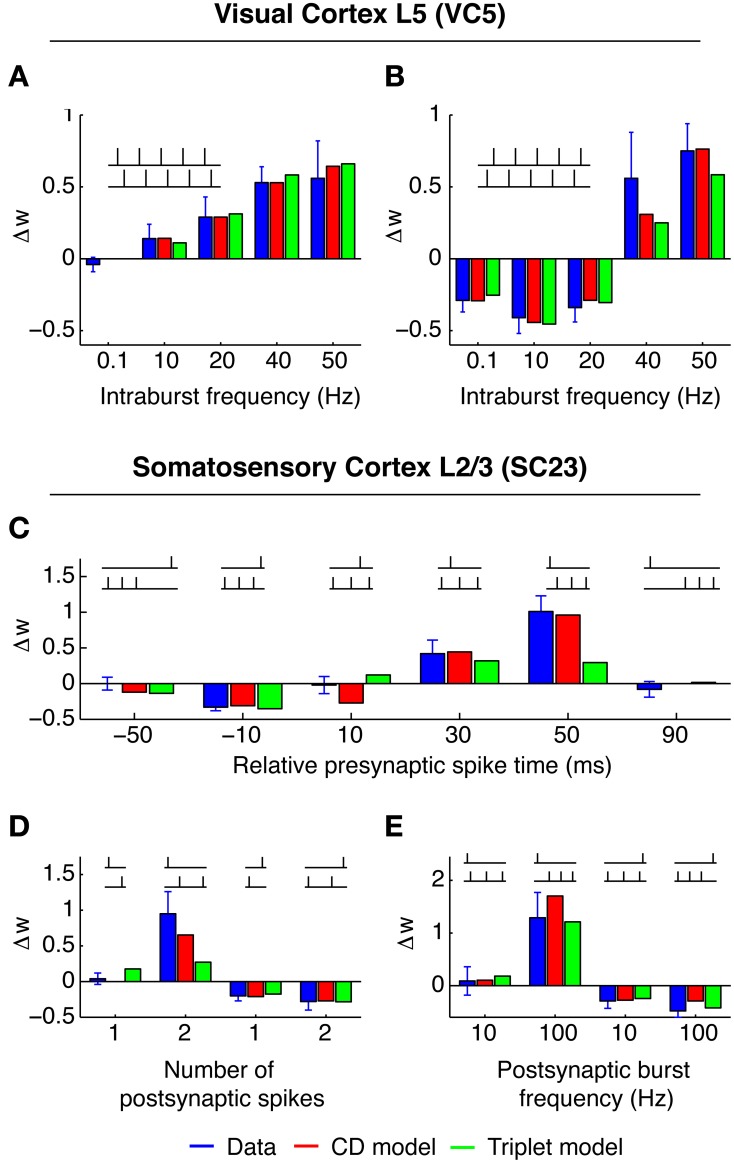
**Best fit of CD model and Triplet model to the data in VC5 (top) and SC23 (bottom)**. Blue bars show experimental weight change ± SEM, red bars show weight change predicted by CD model, and green bars show Triplet model for comparison. Insets show example spike patterns. **(A,B)** Experiments in VC5. **(A)** shows “5–5” bursts with pre- before postsynaptic spiking, **(B)** with order reversed. Both models capture the transition of LTD to LTP with increasing burst rate. **(C–E)** Experiments in SC23. The CD model quantitatively recreates the strong non-linearity of the transition from no change to LTP with the addition of a postsynaptic spike [**(D)**: left vs. second-to-left, and **(C)**: 50 ms].

The experiments were a series of pre-post and post-pre pairs, with fixed timing of 10 ms between the spikes of one pair. The spike pairs were applied with 0.1 Hz (low frequency) 50 times, or organized in “5–5”-bursts (moderate to high frequency), and each burst was induced 15 times. Each burst consisted of 5 spike pairs at intervals of 100, 50, 25, and 20 ms. Two consecutive bursts were 10 s apart. Pre-post spike pairs at low frequency (0.1 Hz) do not change the synapse. We reflect this in the CD model by setting *q*_min_ = 1/(1 + τ_post_/τ_pre_). For post-pre spike pairs of burst frequencies up to 20 Hz, the weight change remained constant (Figure 2B, blue bars). For pre-post pairs, however, potentiation increased with increasing burst frequencies well below 20 Hz (Figure 2A). In the CD model, Δ*w*_−_ is proportional to the integral of the product of the pre- and postsynaptic activity traces *y*_pre_ and *y*_post_. Consequently the time window of interaction is limited by the smaller of the two time constants of decay τ_pre_ = 14 ms and τ_post_ = 42 ms. Because at 20 Hz the distance between each pair is 40 ms, each spike pair remains effectively isolated, and Δ*w*_−_ only depends on the number of spike pairs. For pre-post pairs, however, the outcome is determined by the state of the variable *q* at the time of the postsynaptic spike. The time constant τ_*q*_ is longer (50 ms), which means that as the frequency of spike pairs is increased, at the time of the next postsynaptic spike the variable *q* is still well above baseline level and Δ*w*_−_ and Δ*w*_+_ do not cancel anymore, but Δ*w*_+_ “wins.” For even higher spike pair frequencies (40 and 50 Hz), the spike pairs are so close together that the traces *y*_*i*_ interact. Because *q* has a considerable build-up under these conditions, LTP is favored regardless of spike order. This captures the experimental result that for burst frequencies of 40 and 50 Hz, post-pre pairings potentiate the synapse instead of depressing it.

#### 3.1.2. Area SC23 (Nevian and Sakmann, 2006)

In this study, one presynaptic spike was paired with a train of one to three postsynaptic spikes. Each pattern was repeated 60 times at a repetition rate of 0.1 Hz. Lacking multiple presynaptic spikes the parameters of presynaptic adaptation could not be determined, therefore we set *u*_pre_ = 1 and *c*_pre_ = 0 during the fitting procedure. The pre-post spike pair at Δ*t* = 10 ms does not change the synaptic efficacy, consequently we set *q*_min_ = 1/(1 + τ_post_/τ_pre_). However, LTP is reported for pre-post-post triplets of sufficient postsynaptic burst frequency (≥50 Hz, see Figure 2). This is an example for a “priming” of the synapse. In the CD model, the conditional modulation of LTP by *q* (Equation 11) achieves this. Pre-post pairs induced with low inter pair intervals (5 s and longer) do not change the synapse, but allow LTP to be expressed if a second postsynaptic spike follows the leading pair quickly.

#### 3.1.3. Area HC (Wang et al., 2005)

**Figure 3:**
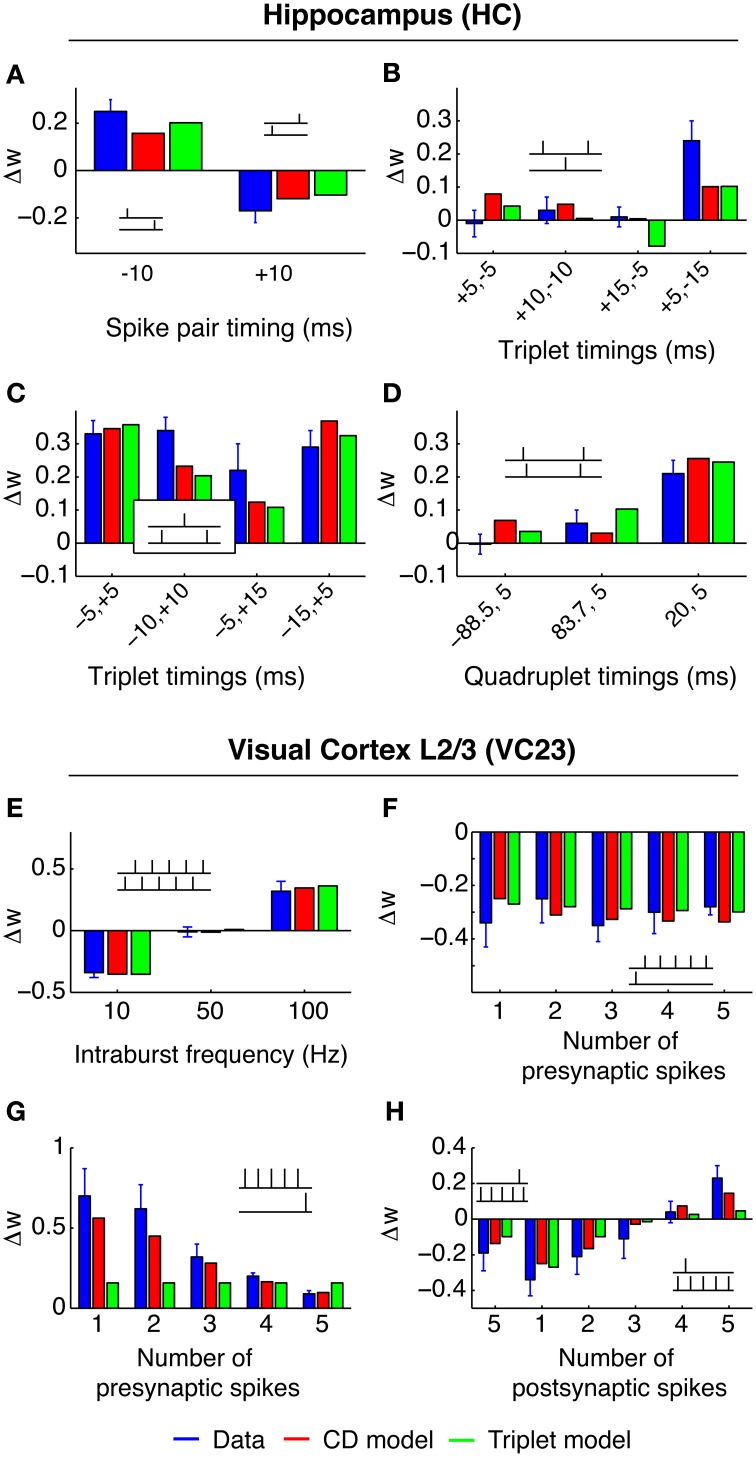
**Best fit of CD model and Triplet model to the data in HC (A–D) and VC23 (E–H)**. In HC, CD model and Triplet model capture the main feature of the results, where pre-post-pre triplets do not change the synapse, while post-pre-post triplets show strong LTP. The +5, −15 triplet (**B**, right) however, can not be reproduced by both models, leading to a relatively large error. In VC23, adaptation is evident in **(G)**. Adding more spikes in front of a pre-post pair decreases LTP, contrary to expectation. The Triplet model has no mechanism which can deal with that.

The experimental setup of this study differs most from the others. The measurements were done in cultured neurons from the hippocampus of rat embryos, compared to neocortical slices of young rats in all other experiments. Also, the spike pattern repetition frequency was higher (1 Hz compared to 0.1–0.2 Hz). The main result of these experiments is the synaptic change in response to several pre-post-pre and post-pre-post spike triplets. For identical timings, spSTDP predicts the same synaptic change for both triplets, because the same post-pre and pre-post pairs occur. But in the experiment, a post-pre-post triplet leads to LTP (Figure 3C), while a pre-post-pre triplet does not change synaptic transmission (Figure 3B). This suggests that the leading postsynaptic spike “primes” the synapse for potentiation, without the need to meet the condition for *q* (Equation 11). In the CD model, this requires a negative threshold ϑ_*q*_ < 0. Compare this to the data in SC23, where the priming is conditional on a pre-post pair, instead of a single postsynaptic spike.

#### 3.1.4. Area VC23 (Froemke et al., 2006)

In this study, several of the features of spike integration were examined. First, “5–5” bursts were conducted similar to the experiment in VC5 (Figure 3A, blue bars). For post-pre spike pairs, LTD converted to LTP with burst frequencies greater than 50 Hz. Second, “*n* − 1” spike patterns were examined to characterize presynaptic adaptation (termed “suppression” in the original article). One to five presynaptic spikes in a burst at 100 Hz were paired with one postsynaptic spike either before or after the presynaptic burst. The result from this experiment can be interpreted such that only the leading presynaptic spike of the burst has a noteworthy influence on synaptic change. In the CD model, this is reflected by strong presynaptic adaptation in the fit to the data. Third and last, “1 − *n*” experiments paired one presynaptic spike with one to five postsynaptic spikes. (Figure 3D). An interesting result is the comparison of the post-post-post-post-pre-post pattern (Figure 3D, left) with the post-pre-post triplet: Both result in the same synaptic change. One possible interpretation is that the leading postsynaptic spikes had little to no influence on the synaptic change. In the CD model, this requires that the threshold ϑ_*q*_ is greater than zero, so that no modulation of Δ*w*_+_ caused by an increase of *q* happens in both spike patterns. If ϑ_*q*_ was smaller than zero, the postsynaptic bursting before the conclusive pre-post pair would lead to a build up of the variable *q*, which in turn would cause Δ*w*_+_ to be strongly upregulated and to “overwhelm” Δ*w*_−_, which was not observed.

The parameters of the best fits to all data sets are shown in Table 3.

**Table 3:** **Parameters of CD model of best fit for each data set**.

**Data set**	**τ_pre_ (ms)**	**τ_post_ (ms)**	**τ^rec^_pre_**	***c*_pre_**	**τ^rec^_post_**	***c*_post_**	***q*_min_**	**τ_*q*_**	***c_q_***	**ϑ*_q_***	***c_w_***	**Error**
VC5	14	42	94 ms	0.7	–	0	0.25	46 ms	1.93	< 0	0.03	0.17
HC	17	34	3 s	0.2	10 ms	0.9	1	20 ms	3.0	< 0	0.009	2.81
SC23	14	42	–	–	20 ms	1	0.25	0.5 s	8.5	0.1	0.018	0.81
VC23	14	42	0.6 s	0.7	0.3 s	0.9	1	0.3 s	6.6	0.1	0.033	0.78

#### 3.1.5. Testing the importance of adaptation

The parameters resulting from the fits show substantial postsynaptic adaptation only for the VC23 data set whereas postsynaptic adaptation is non-existent in VC5 or has very fast recovery reflected by short time constants in SC23 and HC. We therefore tested if postsynaptic adaptation was necessary to explain the data by fitting it a second time with *c*_post_ enforced to be zero; this is effectively switching off postsynaptic adaptation. The resulting errors are given in Table 4. For HC, the increase in error is about 3%, for SC23 the error increases by 23%. This is not a large difference, and it follows that postsynaptic adaptation is not necessary to explain these data sets. In the VC23 data set, the error increases sevenfold. If the CD model is fitted to the data with presynaptic adaptation switched off (*c*_pre_ ≡ 0 for the fitting), the error increases for HC by 26%, for VC5 it more than doubles, and for VC23 the increase is greater than sevenfold.

**Table 4:** **Errors for reduced CD model**.

**Data set**	**Full model**	***c*_post_ = 0**	***c*_pre_ = 0**	**No adaptation**
VC5	0.17	–	0.38	0.59
HC	2.81	2.92	3.55	3.62
SC23	0.81	1.0	–	1.0
VC23	0.78	6.1	7.3	7.8

#### 3.1.6. Comparison with triplet model

The Triplet model (Pfister and Gerstner, 2006) is a model of STDP which is in scope and formulation similar to the CD model. Both extend spSTDP with several non-linearities to account for actual measurements of synaptic changes in complex spike patterns. To gain a relative measure of fitting performance of the CD model, we compare its fit to the different data sets to the ones of the Triplet model. Because in the original article the Triplet model was fitted only to VC5 and HC, we did our own fits to the remaining two data sets, and a re-fit to VC5 (see Materials and Methods). The best fits of the triplet model together with the best fits of the CD model to the different data sets are shown in Figures 2, 3 (green bars). The respective parameters are given in Table 5. For VC5 the change of the experimental protocol in the original article for this data set did not change the resulting error by much, nor the original conclusion that the error is lower with nearest neighbor interactions; the error is 0.51 for all-to-all interactions (parameters not shown). The CD model reaches a lower error (0.17 compared to 0.33), but both models follow the most prominent feature of this data set, the conversion of depression to potentiation with increasing repetition frequency. The preference of nearest neighbor interactions is also found in the fit to VC23, where the error is 25% lower for the model with nearest neighbor interactions compared to all-to-all interactions. An interesting feature of the parameters is that the amplitude of “potentiating” triplet interactions *A*^+^_3_ is negative in VC23. The reason is that the adaptation found in VC23 needs to be accounted for. The triplet model has no (explicit) adaptation, but negative values for *A*^+^_3_ mimic part of the effect.

**Table 5:** **Parameters of best fit for the triplet model**.

**Data set**	**τ_+_ (ms)**	**τ_−_ (ms)**	**τ_*x*_**	**τ_*y*_**	***A*^+^_2_**	***A*^+^_3_**	***A*^−^_2_**	***A*^−^_3_**	**Error**
VC5	17	34	–	38 ms	0	0.049	0.0068	0	0.33
HC	17	34	946 ms	27 ms	0.0061	0.0067	0.0016	0.0014	2.9
SC23	14	42	7.7 s	6 ms	0.006	0.211	0.0004	0.009	1.69
VC23	14	42	2.7 s	2.6 s	0.007	−0.0005	0.0104	0.01	2.78

For the data from SC23 with τ_+_ and τ_−_ taken from VC23 the fit with all-to-all interactions led to the smallest error (1.69 compared to 1.97). The amplitude parameter *A*^+^_3_ is two orders of magnitude greater than the others. This gets outweighted by the time constant τ_*x*_ = 7.7 s, which leads to an high accumulation of trace *r*_2_ that controls the triplet depression. A second fit which allowed τ_+_ and τ_−_ to vary (eight parameters in total) reached a lower error of 1.25, but the resulting time constants of the STDP window have the property that τ_+_ > τ_−_, which is the reverse of what is usually found. For the fit of the “minimal” CD model the error is 1.0, but here only four out of seven parameters were varied: τ_*q*_, *c*_*q*_, ϑ_*q*_ and the scale parameter *c*_*w*_. The other three parameters are kept fixed: τ_pre_ and τ_post_ are set to the values from VC23, and *q*_min_ is determined by the outcome of the pre-post spike pair experiment.

### 3.2. Synaptic theta-susceptibility in spSTDP

**Figure 4:**
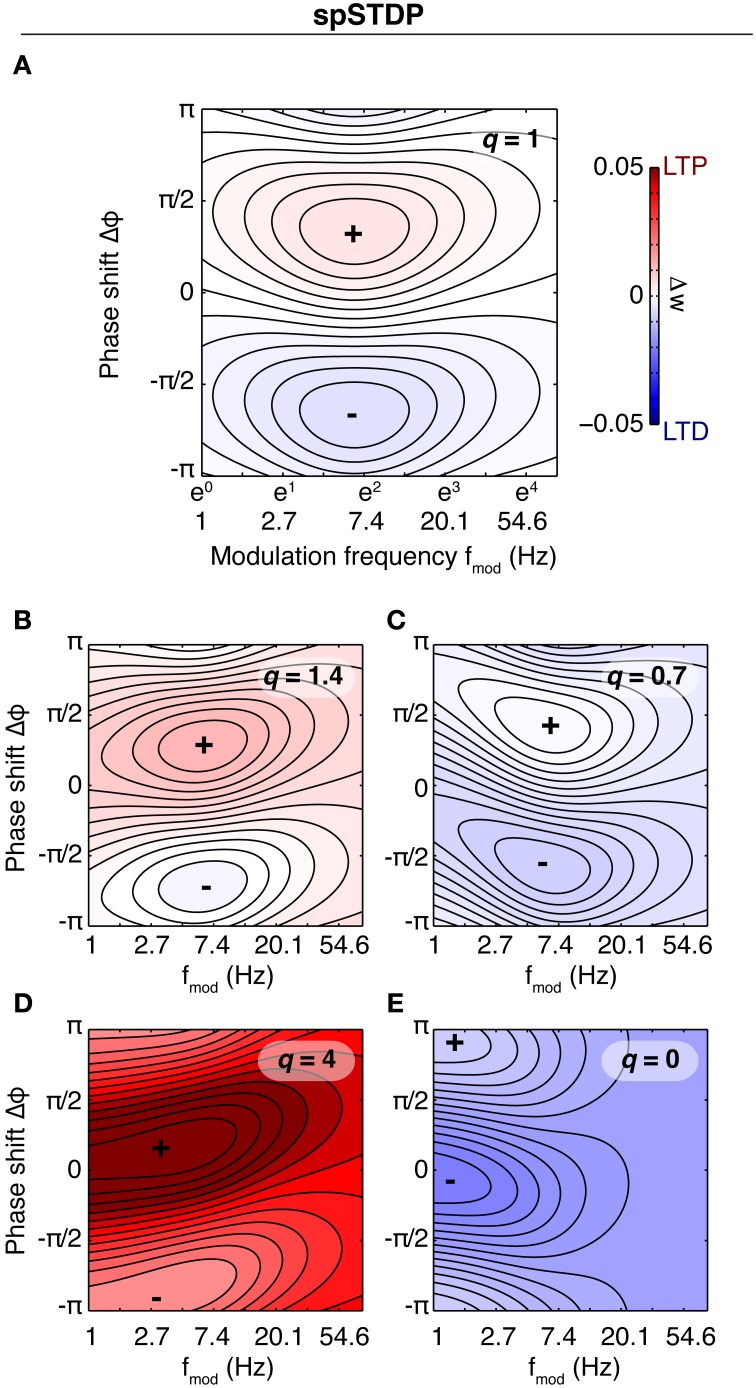
**Rate of weight change in spSTDP**. These plots show the rate of weight change for simple spSTDP (Time constants taken from VC23). Contours mark lines of same weight change. Colors indicate positive (red) or negative (blue) weight change, see colorbar. Colorbar is the same for every plot. Plus and minus signs mark maximum and minimum, respectively. Weight change is a function of modulation frequency *f*_mod_ and phase shift Δφ [see Equation (31)]. Δφ > 0 indicates a phase where presynaptic leads postsynaptic activity. Baseline firing rate is unspecified (see text). **(A)** Balanced spSTDP (*q* = 1). Maximal and minimal weight change occur at *f*_mod_ ≈ 6 Hz, while for very low and very high modulation frequencies the weight change decays to an average value (zero in this case), regardless of phase shift. We term this the band-pass property of the synapse. **(B,C)** spSTDP with a moderate bias toward LTP (*q* = 1.4) or LTD (*q* = 0.7). The bias converts the weight change to LTP or LTD almost everywhere, however, the band-pass property is mostly preserved. **(D,E)** spSTDP with a strong bias toward LTP or LTD. The rate of weight change shows a strong dependence on phase shift even for lowest oscillation frequencies instead of a decay back to an average value. Therefore, the synapse acts as a low pass filter in both cases.

Several studies found that the presence of oscillations with high theta power or large scale theta synchronization in EEG or LFP enhance learning. (Mölle et al., 2002; Seager et al., 2002; Guderian et al., 2009). A possible explanation for these findings could be an underlying filter property of the single synapse, i.e., synaptic change depends on oscillating activity of both neurons in a way specific to the oscillation frequency. To investigate this, we assume a very simple model system: Two connected neurons fire stochastically with vanishing spike-spike correlations, i.e., correlations induced when a presynaptic neuronal activity changes the probability of postsynaptic spikes. Theta oscillations found in EEG or LFP are modeled by periodic sinosoidal modulation of the baseline neuronal activity. Neurons fire spikes with an average rate that is independent of the modulatory rate. Such a modulation could be e.g., induced by periodic inhibition delivered by external sources. As a first step we investigate the filter properties of spSTDP. In Figure 4 we display the rate of weight change as a function of modulation frequency *f*_mod_ and phase difference Δφ for five different values of q, corresponding to differently biased STDP. For a given *f*_mod_ in balanced spSTDP the plot shows that synaptic change shows the greatest difference between minimum and maximum (malleability or susceptibility) in the theta range (4–10 Hz). For high or low frequencies the change decays back to zero. An analytical calculation (see Appendix) shows that the maximally effective modulation frequency lies at

(22) $$f_{\text{max}}=\frac{1}{2\pi \sqrt{\tau_{\text{pre}}\tau_{\text{post}}}}.$$

The frequency of maximum efficiency is a function of the time constants of the STDP window. For the parameters from VC23 used in Figure 4, τ_pre_ = 14 ms and τ_post_ = 42 ms, *f*_max_ = 6.56 Hz. For the time constants from HC, τ_pre_ = 17 ms, τ_post_ = 34 ms, *f*_max_ = 6.62 Hz. In general, for physiological parameters the most effective frequency lies in the theta band.

This is a band-pass filter property, which discards too slow or too fast oscillations, and uses intermediate oscillation frequencies as signals for synaptic change. This is contrasted by strongly biased spSTDP (Figures 4C,E). Here, the region of maximal phase dependency of synaptic plasticity on relative phase (i.e., the region of high susceptibility) is not cut off anymore for low modulation frequencies. The synapse acts as a low pass filter.

### 3.3. Synaptic susceptibility in non-linear models of synaptic Plasticity

We chose five different models of synaptic plasticity to compare the filter properties between them. First we examine the effects of extending spSTDP with realistic non-linearities, with the CD model and the Triplet model. Furthermore, we examine three calcium-based models. The first one is the model of Shouval et al. (2002) (“Shouval model” in the following), which introduced a formalization of the calcium control hypothesis. This hypothesis states that moderately elevated levels of calcium in the postsynaptic spine lead to synaptic depression, while high levels lead to potentiation. The goal is then to model the calcium dynamics at the synapse. For reference, we repeat the equations of the Shouval model in the appendix. The second model is an extension of the Shouval model with pre- and postsynaptic adaptation and presynaptic facilitation (Cai et al., 2007, Cai model). The adaptation is shared with the CD model. The third calcium model was developed by Graupner and Brunel (2012) (“Graupner model”). This model makes similar use of the calcium control hypothesis, however, it combines it with a bistable synapse model (Graupner and Brunel, 2007). All models start from biologically plausible first principles and derive the STDP window as a consequence. Also, each model has inherent weight limits, which force us to change the induction protocol for them (see Methods). For all models, we do the analysis with all available parameter sets.

We constrain ourselves to models which use only spikes (spike times) as relevant signals, and derive all relevant variables from them. There exist models which explicitly take subthreshold neuronal dynamics into account (see e.g., Clopath et al., 2010). Although this type of model is potentially more accurate at describing experimental results, it has to make specific assumptions about neuronal dynamics, which we want to avoid here.

**Figure 5:**
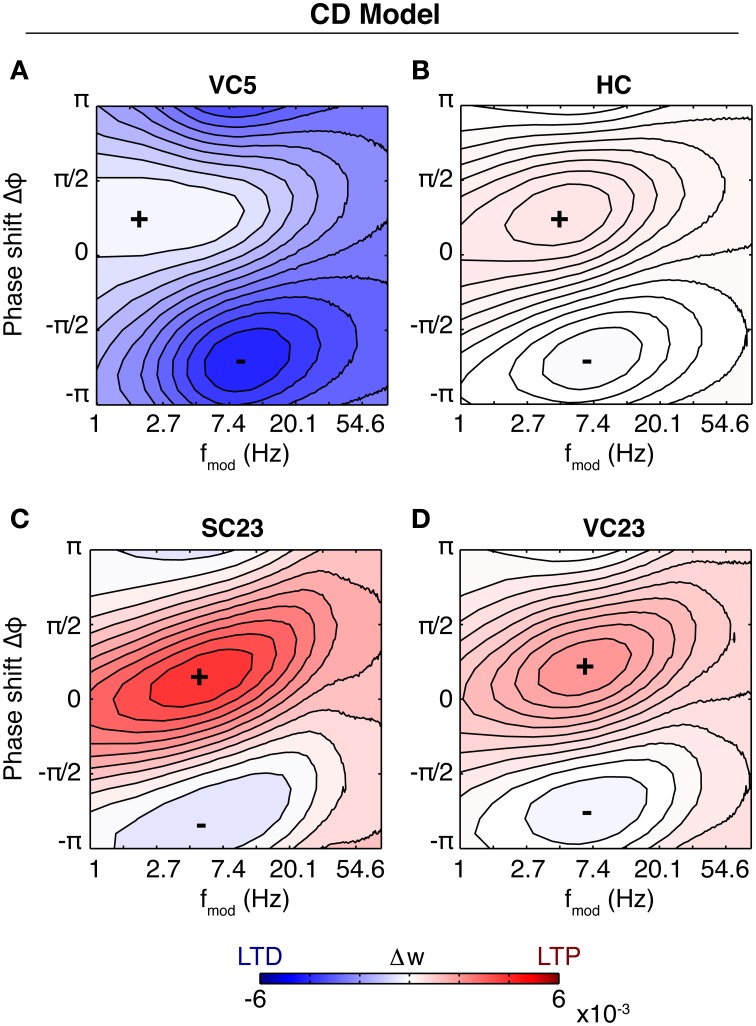
**Susceptibility of the synapse to theta oscillations in the CD model**. Shown is the rate of weight change as a function of phase shift and modulation frequency (similar to Figure 4). Baseline firing rate is 5 Hz for each plot. Same colorbar for all plots. **(A)** Parameters of fit to VC5. The synapse depresses for all modulation frequencies and phase shifts. **(B)** Parameters of HC. The synapse is biased toward potentiation. Maximal potentiation occurs for zero or small positive phase shifts at a modulation frequency of ~2–10 Hz. The result is similar for SC23 [**(C)**, with τ^rec^_pre_ = 0.6 s, *c*_pre_ = 0.7 from VC23] and VC23 **(D)**, with slight variations in magnitude and size of the zone of maximal LTP. Comparison with Figure 4 shows that spSTDP with a moderate bias toward LTP exhibits very similar characteristics.

For the CD model, the rate of weight change as a function of modulation frequency and phase difference is shown in Figure 5. Because the data in SC23 can not give information about presynaptic adaptation, we use τ^rec^_pre_ and *c*_pre_ from area VC23 instead. Three of the plots show a very similar behavior. The weight change is positive almost everywhere, and the zone of maximal LTP depending on modulation frequency is tilted such that for a modulation frequency of 1 Hz highest potentiation occurs at Δφ ≈ 0. A comparison with Figure 4 shows that in the case of a slight bias toward LTP (*q* = 1.4) the picture looks similar. In the parameters for VC23 and HC, *q*_min_ = 1, which means that *q*(*t*) ≥ 1 and the requirement for potentiation-biased STDP is fulfilled. In SC23, the STDP window is biased toward LTD (*q*_min_ = 0.25), however, the parameters for the activation *q* show that contributions to it are strong (*c*_*q*_ = 8.5) and last long (τ_*q*_ = 0.5 s). Therefore the bias toward LTP results from the baseline activity. The rate of weight change in VC5 deviates strongly from this, it shows an asymmetry between maximal and minimal weight change, and is strongly biased toward LTD.

**Figure 6:**
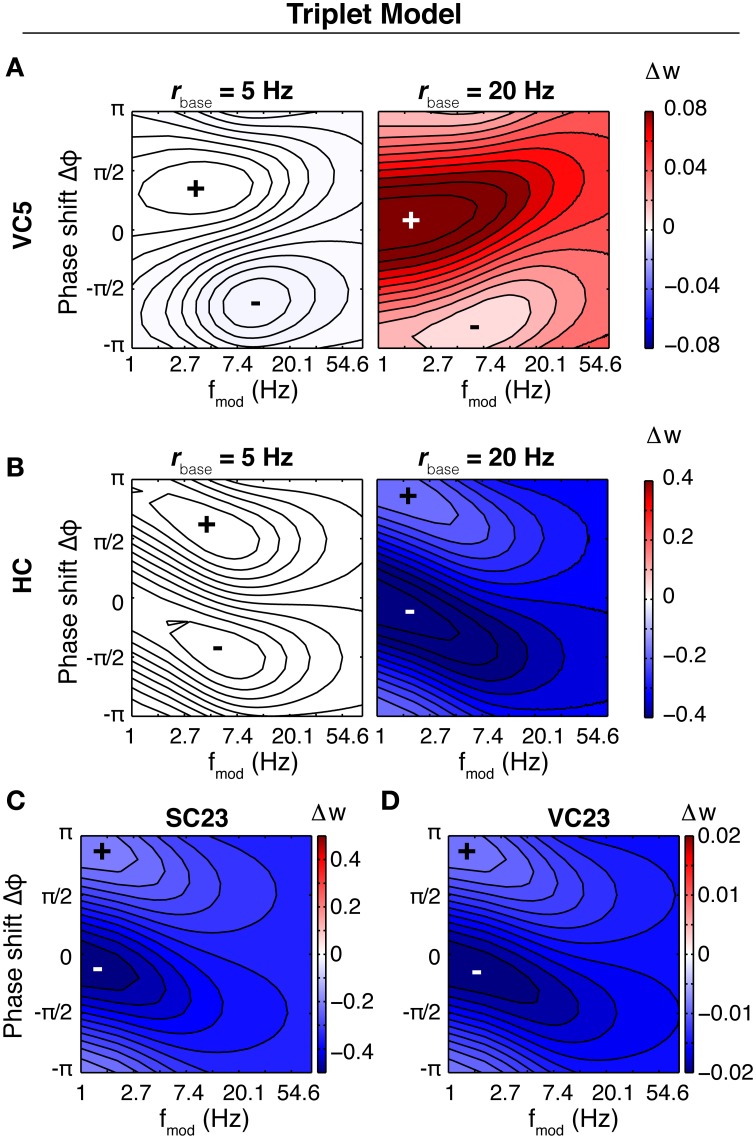
**Susceptibility of the synapse in the Triplet model**. Shown is the rate of weight change of the synapse as a function of *f*_mod_ and Δφ in the Triplet model, with each of the parameter sets and with different neuronal baseline firing rates. In contrast to Figure 4 the colorscale is separate for each plot. **(A)** Area VC5, left: 5 Hz average firing rate, right: 20 Hz firing rate. At 5 Hz, the weight change is negative everywhere (not visible because of the order of magnitude). Changing the firing rate to 20 Hz leads to LTP everywhere. **(B)** Area HC, at 5 and 20 Hz average firing rate of the both neurons. The weight change is negative for both conditions. **(C,D)** Areas SC23 and VC23 at a baseline firing rate of 5 Hz. With both parameter sets, the weight change is purely negative.

The characteristics of the susceptibility in the Triplet model are different from the CD model (Figure 6). In three parameter sets (HC, SC23, VC23), the weight change is depression only, and the tilt of maximal weight change (closest to zero) is inverted compared to the CD model. None of these three parameter sets shows a pronounced susceptibility specific to a certain frequency range. Increasing the baseline rate does not change the weight change qualitatively, but rather scales it up (Not shown for SC23, VC23). Comparison with spSTDP (Figure 4; *q* = 0.7) suggests that the respective parameters are biased toward LTD. Like in the CD model, area VC5 stands out. At 5 Hz the weight change is the same as in the CD model. For an increased baseline rate of 20 Hz, it changes from depression to strong potentiation, as comparison with Figure 4C shows.

**Figure 7:**
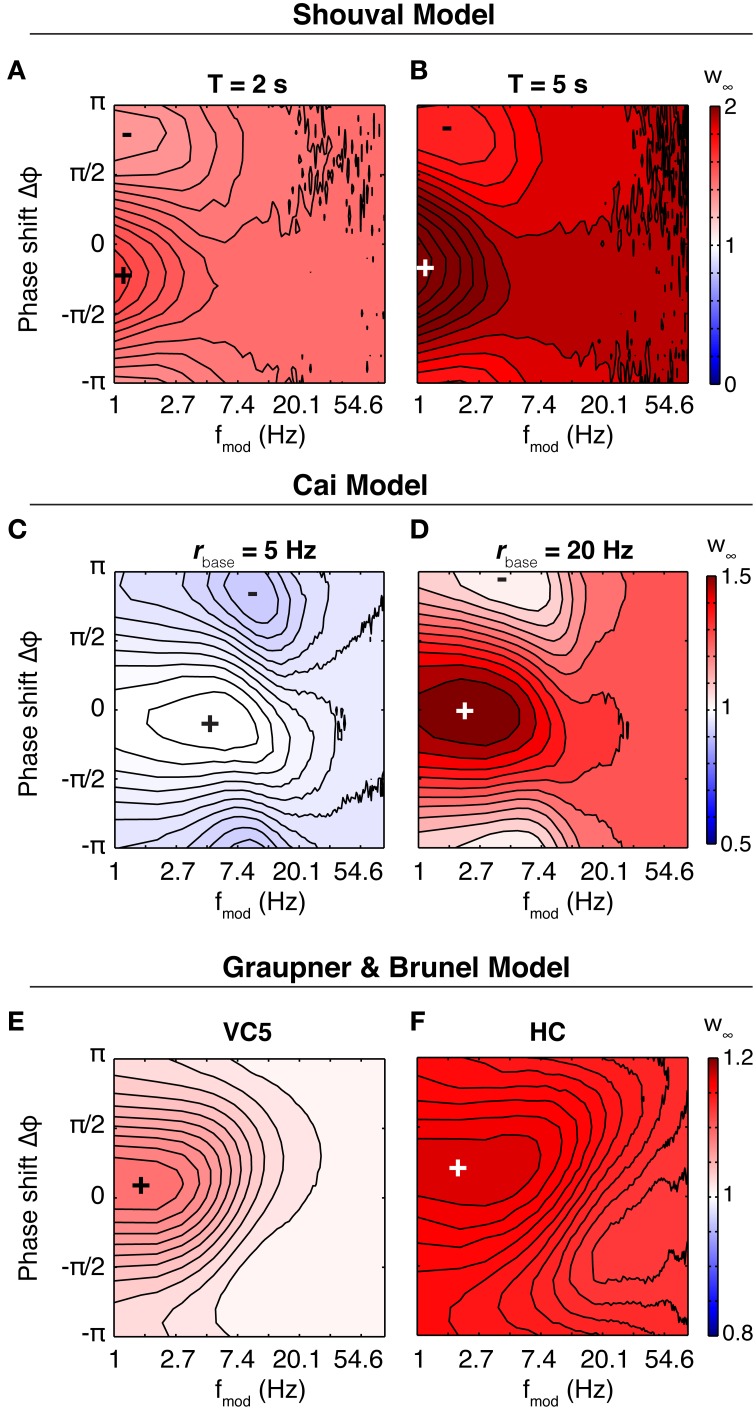
**Synaptic susceptibility in the calcium models**. Top row: Final synaptic weights after 2 s **(A)** and 5 s **(B)** of stimulation in the Shouval model. Baseline firing rate is 5 Hz. The synaptic weight shows a phase dependence only for lowest modulation frequencies. The result is similar for other baseline firing rates (not shown). Middle row: Final weights in the Cai model. Baseline firing rate is 5 Hz **(C)** and 20 Hz **(D)**, duration of theta modulated firing is 10 s. At 5 Hz, the synapse shows a preference for modulation in the theta range (centered at ~5 Hz). Neither of the two other models does something similar. Bottom row: Final synaptic weights in the model of Graupner and Brunel after 5 s of stimulation, with parameters fitted to VC5 **(E)** and HC **(F)**. Baseline firing rate is 10 Hz. Similar to above, the synapse reacts strongest to slow and slowest oscillations. Synapses in calcium models are low-pass filters.

In the Shouval model, the susceptibility depends little on the parameters of the induction protocol (stimulation time 2 or 5 s, baseline firing rates different from 5 Hz (not shown), Figure 7). The synapse potentiates for all parameters, and shows a phase dependence for slow oscillations (<3 Hz). However, the difference between maximum and minimum weight is small. The situation is similar in the Graupner model: In VC5 the phase dependence of weight change reaches into the theta band, in HC up to 20 Hz (Figure 7). The synapse, however, shows no band-pass properties. In contrast to the other two models, in the Cai model the synapse is susceptible to oscillations in the theta band. However, as the baseline firing rate is increased, the synapse shifts to a low pass filter.

### 3.4. Contributions to theta susceptibility

**Figure 8:**
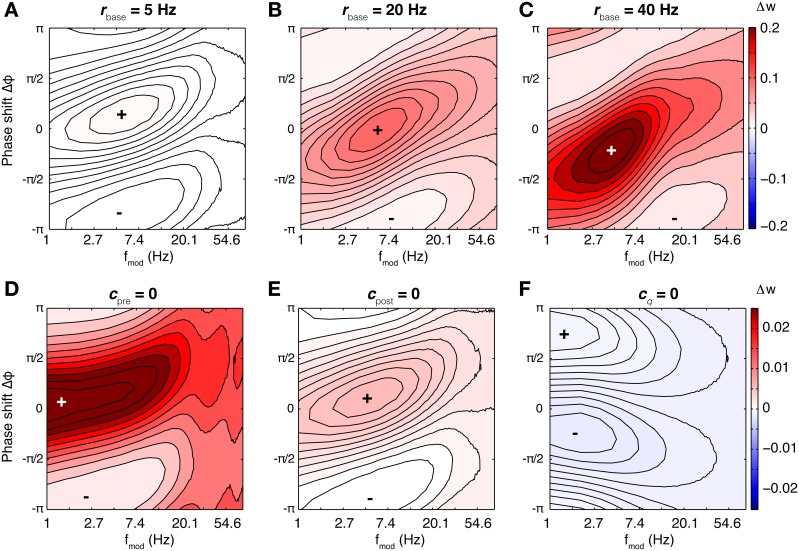
**Susceptibility in the CD model under varying conditions**. For the base parameters for SC23 we repeated the simulations to compute the rate of weight change under different conditions. Top row: Weight change with increasing baseline firing rate (**A**: 5 Hz, **B**: 20 Hz, and **C**: 40 Hz). At low firing rates, the synapse behaves similar to linear spSTDP; maximal LTP occurs at Δφ ≈ π/6 (compare Figure 4). With increasing firing rates, maximal potentiation also increases, and maximal LTP shifts toward slightly negative phase shifts (20 Hz: Δφ ≈ 0 40 Hz: Δφ ≈ −π/4), while being centered at *f*_mod_ ≈ 5 Hz. Bottom row: Influence of model constituents on susceptibility. Neurons fire at an average rate of 5 Hz. **(D)** CD model without presynaptic adaptation. The synapse is strongly sensitive to Δφ, however, there is no lower bound on *f*_mod_. **(E)** Without postsynaptic adaptation. The susceptibility of the synapse is very similar to that of the undisturbed model (compare **A**). **(F)** Without activation *q*. The weight change is negative everywhere, and the synapse is not susceptible for some intermediate *f*_mod_. Plots **(D,F)** change only slightly with increasing baseline firing rates. We conclude that of the model constituents, postsynaptic adaptation is not necessary to explain theta susceptibility in the non-linear regime.

The CD model is the only model of synaptic plasticity tested here which retains a susceptibility specific for a certain frequency range beyond the linear regime (low firing rates). To illustrate the behavior in the non-linear regime, we computed the rate of weight change for different baseline firing rates for the parameter set of SC23 (Figures 8A–C). At low firing rates, the susceptibility is very similar to that of spSTDP with a moderate bias toward depression (see Figure 4B). One could expect that with increasing firing rate, the bias simply gets stronger until the weight change is similar to Figure 4C. However, the specific susceptibility gets slightly more pronounced, and the maximum of potentiation moves toward (and for very high firing rates beyond) zero phase shift. This effect is stronger for low modulation frequencies (≈3 Hz, Figure 8C). Interestingly, this property resembles the experimental observation that presynaptic stimulation repeatedly delivered at the peak of a theta oscillation potentiates the synapse, while stimulation at the trough depresses it (Hyman et al., 2003).

**Figure 9:**
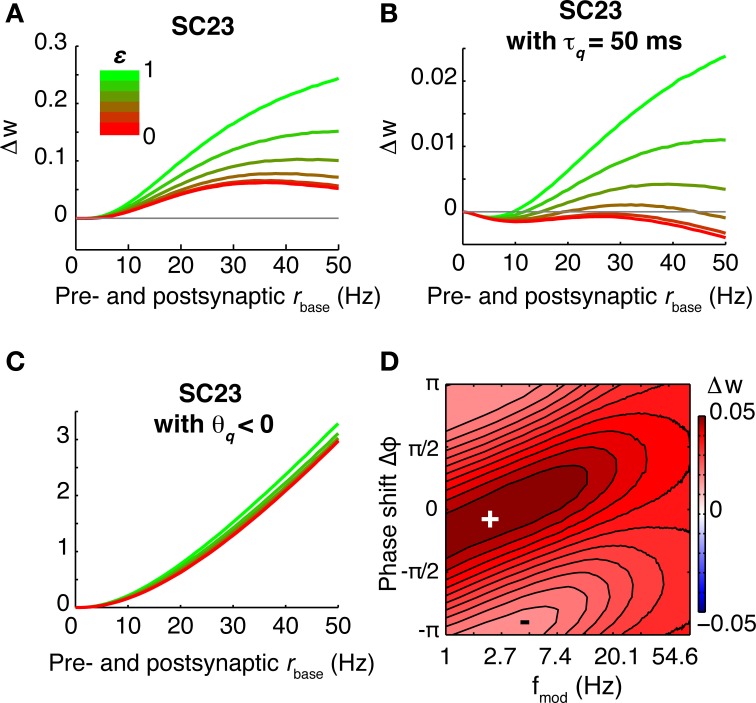
**Effect of different oscillation amplitudes, and negative threshold ϑ_*q*_. (A–C)** Show the rate of weight change as a function of common pre- and postsynaptic baseline firing rate for Δφ = 0, *f*_mod_ = 5 Hz and different oscillation amplitudes ε ∈ [0, 1]. **(A)** Parameters from SC23. Because of the strong contributions to *q*, the weight change is positive for all firing rates. Introducing the theta modulation increases weight change even more. **(B)** Parameters from SC23 with shorter time constant of activation: τ_*q*_ = 50 ms instead of 500 ms. This manipulation increases the error from 0.81 to 1.1. Under this condition, imprinting theta oscillations on the neurons alters LTD to LTP. **(C)** Same as **(A)**, but with ϑ < 0. Removing the threshold from the activation reduces the sensitivity to theta oscillations. **(D)** Weight change as a function of Δφ and *f*_mod_, with parameters as in **(C)**. The weight change is similar to spSTDP with strong bias toward LTP (Figure 4C). The maximal potentiation at 1 Hz modulation frequency occurs at negative phases.

Next, we investigate the effect of increasing oscillation amplitude on the synapse. We keep the modulation frequency fixed at 5 Hz with Δφ = 0, and vary the oscillation scaling parameter ε. In the case of the SC23 parameters (Figure 9A), the synapse potentiates for constant (unmodulated) firing for baseline rates greater than 5 Hz. Theta oscillations in the firing rates simply lead to an upscaling of this potentiation. We do the same analysis for an altered set of parameters (Figure 9B). We change τ_*q*_ to 50 ms instead of 500 ms. This tones down the activation and therefore potentiation, however, it affects the fitting error only slightly (increase from 0.81 to 1.1). In this case, the weight change for constant firing is negative everywhere. Introducing a periodic modulation then increases weight change, turning depression into potentiation, but only for baseline firing rates greater than 5 Hz.

To elucidate what parts of the model are responsible for this susceptibility, we did the analysis for confined versions of the CD model. The resulting weight change as a function of modulation frequency and phase difference is shown in Figures 8D–F, where we removed presynaptic adaptation, postsynaptic adaptation and activation *q*, respectively. The weight change shows that presynaptic adaptation as well as activation are both important for theta susceptibility. Removing either one results in a low pass filter synapse. The link between these two variables is the threshold ϑ_*q*_, as Figure 9D illustrates. Here, we show the weight change for the full model and parameters of SC23, except that ϑ_*q*_ < 0. As a consequence, the synaptic susceptibility has no cutoff for low frequencies anymore. Interestingly, removing the threshold removes large part of the sensitivity to increasing oscillation amplitude (Figure 9D), which further underlines the importance of the interplay of presynaptic adaptation and activation for theta susceptibility.

## 4. Discussion

We presented a new phenomenological model for dynamic synaptic plasticity, which unifies several experimental results in one framework. We analyzed the filter properties of this model, and compared them to a range of other models. We found that the CD model has unique properties which tie in with experimental findings on the connection of theta oscillations and memory formation, thus providing a mechanistic link between synaptic plasticity and the beneficial nature of theta-band oscillations for learning.

### 4.1. Interpretation of model components and parameters

Although most of the components of the CD model are only loosely guided by biophysical considerations, it is possible to relate them to specific perisynaptic processes, and to envision experiments for a more direct parameter estimation.

The spike traces *y* are very similar to the dynamics of bound glutamate at postsynaptic receptors and calcium dynamics in the synaptic bouton, which are essentially low-pass filtered action potentials. Due to the differential Hebbian learning rule at the core of the CD model, the decay constants of the spike traces determine the shape of the classical exponential STDP window; therefore, they can be directly estimated from varying the timing of a single pre- and postsynaptic spike pair.

The adaptive suppression *u*, which leads to a sublinear summation of synaptic change, has a dynamics reminiscent of presynaptic short-term depression (Tsodyks and Markram, 1997; Tsodyks et al., 1998). Its parameters can be determined by measuring the change of the synapse in response to adding leading presynaptic (postsynaptic) spikes to a pre-post (post-pre) spike pair and comparing the experimental result to the prediction of a spike pair model. Presynaptic short-term depression is a well-understood phenomenon, which has been shown to be present in many different cell types (Zucker and Regehr, 2002). Interestingly, the results of the fits of the reduced model indicate that short term depression considerably influences synaptic change and should be taken into account in quantitative models of synaptic plasticity. Some synapses show facilitation as well, and the CD model can easily be accommodated to include this also by adding one equation in a manner similar to Tsodyks et al. (1998). On the postsynaptic side, however, the mechanism behind the adaptive process has been studied much less (Froemke et al., 2006; Gasparini, 2011); the details of the formalization will possibly have to be adapted as soon as more quantitative data becomes available.

The conditional activating variable *q* is a coincidence detector, which primes the synapse for supralinear potentiation. An interpretation is that *q*, if dependent on the presynaptic trace, reflects the calcium trace from NMDA receptors, which rely on the coincidence of glutamate binding and postsynaptic depolarization to lift the Mg^2+^ block (Clarke and Johnson, 2006). A negative threshold could mean that less calcium is needed for induction of LTP, or influx of calcium through voltage dependent calcium channels is sufficient for an elevated trace. The relaxed state value *q*_min_ on the other hand tunes the balance of LTP and LTD at the synapse. Under certain conditions, like in SC23, a pre-post pair leads to calcium influx which does not exceed the threshold for induction of LTP. A second postsynaptic spike added after the pair then “rides” on an elevated level of calcium, and the summed calcium contributions exceed this threshold. To estimate the parameters of activation with known postsynaptic adaptation, trailing postsynaptic spikes can be added to a pre-post-pair, with an additional post-pre-post triplet to find the sign of the threshold.

### 4.2. Relation of the CD model to other models

In the last decade, a number of models for synaptic plasticity in response to spiking activity have been developed. Some of them are extensions of spSTDP, like the Triplet model, others are grounded on more biophysically plausible considerations, like the calcium models in general. The only model we know of which was fitted to the same four data sets as the CD model here is the recent calcium model by Uramoto and Torikai (2013). They used a different way to calculate the error of their fit, as they did not normalize the experimental results with the standard error of the mean (SEM). We repeated the calculation of the error with normalization by SEM, with the parameters given in the original article. The resulting errors are 0.85 for VC5, 2.4 for HC, 0.27 for SC23, and 27.39 for VC23. See Tables 3, 5 for comparison. The last value mostly results from the omission of the “1−5 × pre-post” experiments by the authors [Figure 4D in the original article of Froemke et al., (2006)], although the error without these experiments is still 9.2. These experiments most prominently highlight the role of presynaptic adaptation: Adding more presynaptic spikes in front of the pre-post pair reliably decreases the magnitude of synaptic potentiation. The Uramoto model has no mechanism which results in less postsynaptic calcium (less LTP) with increasing number of presynaptic spikes, a property shared with the Triplet model, Graupner model and Shouval model. Our quantitative analysis in the CD model showed that in the data sets where applicable (SC23 used only one presynaptic spike in each induction pattern), adding presynaptic adaptation considerably improves the error. However, presynaptic (and postsynaptic) adaptation can easily be implemented in all these models, for example by introducing Equation (9). Different formalizations are realized by Cai et al. (2007) and Kumar and Mehta (2011). We propose that adaptation is a mechanism that should in general be considered for the quantitative modeling of synaptic plasticity.

Among the existing phenomenological models of STDP, the Triplet model is the one most similar to our CD model. Both show similar fitting performance on visual cortex layer 5 and hippocampal data sets. Although it lacks true adaptation, two properties of the Triplet model partially mimic it: (1) with nearest neighbor interactions a spike causes the trace to attain a certain, constant value (from where it relaxes back). If the trace still is greater than zero, the impact of the subsequent spike is reduced; (2) a negative value for the triplet interaction *A*^+^_3_ works in the opposite direction of the normal spike pair interaction, leading to a sublinear summation of potentiation. In total, the CD model reaches a lower error than the Triplet model on all data sets, in SC23 and VC23 by a considerable margin. This suggests that the CD model generalizes better than the Triplet model. In addition, the components of the CD model have a more straightforward interpretation.

### 4.3. Theta susceptibility in different plasticity models

We investigated the filter properties of synaptic plasticity in a range of different models. For balanced spSTDP, we can give an explanation of the origin of the susceptibility to intermediate neuronal activity oscillation. spSTDP is equivalent to the formulation as differential Hebbian learning, Equations (2), (3). The traces *y*_*i*_ are driven by the spike trains *x*_*i*_, and they “smear” out the spike over time, which is basically the action of a low-pass filter. However, the weight change is proportional to the product of the presynaptic trace and the temporal derivative of the postsynaptic trace, *ẏ*_post_. A temporal derivative accentuates (fast) changes, and its effect is similar to a high-pass filter. The result is a band-pass filter with an oscillation frequency of maximal efficiency given by Equation (22). If a moderate bias is introduced (see Figures 4B,C), the basic finding is distorted only slightly.

For non-linear models of synaptic plasticity that are based on spSTDP, the picture in general is similar as long as the average firing rate stays low, which keeps the dynamic equations in the linear regime. If the firing rate gets high enough that spikes in a neuron start to interact with each other, non-linear interactions will start to distort the susceptibility. For a mean firing rate of 5 Hz, the CD model stays in a near-linear regime, while in the Triplet model non-linear effects abandon susceptibility. Interestingly enough, with the right parameter choice the non-linearities in the CD model retain a band-pass behavior similar to the linear regime (Figures 8A–C). The susceptibility in the non-linear regime depends on the interplay of *u*_pre_ and *q* with a threshold for activation ϑ greater than zero. The action of presynaptic adaptation is to suppress *y*_pre_ for sustained constant firing of the presynaptic neuron. In fact, a mean field calculation shows that for parameters in VC23 in equilibrium and in the limit of high firing rates 〈*y*^∞^_pre_ 〉 = 0.033 < ϑ_*q*_. However, with oscillating neuronal firing, *y*_pre_ reaches a maximum early during the rise of the rate. The condition on the activation *q* leads to maximal increase if the postsynaptic firing rate is maximal at the same time. Therefore, *q* is maximal for slightly negative phases, which leads to the observed phase shift in the transition to the non-linear regime (increasing baseline firing rates).

We found that in contrast to spSTDP-based models, the synapse in the two calcium models without adaptation is simply a low pass filter, and prefers oscillations of both neurons that are in phase. This is due to the extensive low-pass filtering in the dynamical equations in these models. In both models, the contributions to the calcium concentration are low-pass filtered spike signals, which get low-pass filtered again in the calcium dynamics. As the calcium concentration depends on the sum of pre- and postsynaptic contributions, it is not surprising that maximal LTP occurs close to zero phase and slow oscillations. The Cai model is an example of a calcium model with synaptic short term dynamics. Here, the synapse shows a susceptibility to oscillatory modulation in the theta band, if the average firing rate is sufficiently low. Interestingly, our result seemingly conflicts with previous results. In the study of Kumar and Mehta (2011), it was shown that with the Shouval model the STDP window shows maximal malleability, that is the difference between maximal and minimal synaptic change, if the spike pairs are delivered with a repetition frequency of 5–15 Hz. This is very similar to our definition of susceptibility, where there exists a region of *f*_mod_ with pronounced and maximal difference between maximal and minimal weight change. However, in our study neuronal firing rate and oscillatory modulation (*f*_mod_) are decoupled, while in the aforementioned study they are equal. Furthermore, we investigated the malleability under the condition of stochastic spiking. Here, spSTDP based models in a near linear regime show a preferred window of modulation frequency, while calcium based models prefer slow oscillations.

### 4.4. Theta susceptibility in synaptic plasticity

Theta band (4–8 Hz) oscillations of both cortical (Landfield et al., 1972) and hippocampal (Berry and Thompson, 1978) local field potentials have been associated with memory processes early on. Later studies extended these findings across species and spatial scales, i.e., from intracellular membrane potential fluctuations in the rodent hippocampus (Harvey et al., 2009) to intracranial recordings in monkey cortex (Liebe et al., 2012) and extracranial EEG in humans (Kahana et al., 2001). Despite being observed throughout the brain, theta band oscillations appears to be generated by a network of hippocampal oscillators (Colgin, 2013), which is then transferred into cortical areas.

Although many studies have established a correlation between activity in the theta frequency band, so far no direct explanation for how theta rhythms influence memory processes has been found (Colgin, 2013). Some studies (Berry and Thompson, 1978; Seager et al., 2002; Nokia et al., 2008) report that the indicator for learning success is the increased oscillation amplitude before the onset of a trial. In other words, theta can be present without being linked to a certain task and still be beneficial. Others find that bursts delivered at theta frequency are optimal for induction of LTP (Larson et al., 1986), and LTD and LTP are inducted by bursting at different phases of a background theta oscillation: Presynaptic bursts at the peak of the oscillation potentiate the synapse, while bursts at the trough lead to synaptic depression (Pavlides et al., 1988; Hyman et al., 2003). In humans though, the situation is not as clear. Some studies find that increased theta power predicts learning success (Sederberg et al., 2003; Guderian et al., 2009; Lega et al., 2012), others emphasize theta synchronization and sometimes find decreased theta power (Mölle et al., 2002; Burke et al., 2013). One experimental study found that in successful learning single neurons show enhanced phaselocking to a background theta oscillation in the LFP, with a wide distribution of specific phase relations to this theta oscillation (Rutishauser et al., 2010). All these observations make it very likely that theta oscillations play a constructive role in the formation of memory.

The synaptic filter properties of several plasticity models reported here provide an explanation, as they endow the synapse with a susceptibility that is specific to oscillations in the theta range. This susceptibility does not rely on precise spike timing, i.e., a fixed phase relation of repeated spikes to an ongoing background theta oscillation. The distinction between the linear (similar to spSTDP) and non-linear regimes we found in the models makes two different scenarios likely of how theta susceptibility plays a role in learning. With low baseline firing rates [<10 Hz, reported in Rutishauser et al. (2010)] and a wide distribution of pairwise relative phases the synaptic changes are also expected to show a wide distribution of values. In a neuronal population firing at higher baseline rates the interplay of presynaptic adaptation and conditional activation shifts the phase requirement for strongest LTP to synchronous (phase zero) oscillation. How can a synapse capitalize on that? The scenario in Figure 9 provides a possible answer. The synapse depresses uniformly for neuronal constant firing. Introduction of theta band oscillations (5 Hz) shift up the weight change, but only for elevated baseline firing rates. The result is Hebbian learning (“those who fire together wire together”), as synapses between neurons which get no external excitation slightly depress. In this scenario, theta oscillations can be present before external stimulation, preparing the synapses for learning correlations of neuronal firing.

### Conflict of interest statement

The authors declare that the research was conducted in the absence of any commercial or financial relationships that could be construed as a potential conflict of interest.

## Appendix

### The model of Shouval et al. (2002)

We decided to include a full description of the model from (Shouval et al., 2002) here. We found the description given in the original article not sufficiently clear.

In this model, spikes from either the pre- or postsynaptic neuron influence the postsynaptic membrane potential with an EPSP (pre) or a back propagating action potential (bAP; post), which add linearly:

(23) $$V(t)=\int_{-\infty }^{t}\text{EPSP}\left(t-t^{\prime }\right)x_{\text{pre}}\left(t^{\prime }\right)dt^{\prime }+\int_{-\infty }^{t}\text{bAP}\left(t-t^{\prime }\right)c_{\text{post}}\left(t^{\prime }\right)dt^{\prime }$$

$$\begin{array}{l} \text{EPSP}(s)=\Theta (s)A_{\text{EPSP}}\left(e^{-s/\tau_{s}^{\text{EPSP}}}-e^{-s/\tau_{f}^{\text{EPSP}}}\right)\\ \text{ bAP}(s)=\Theta (s)A_{bAP}\left(I_{f}^{\text{bAP}}e^{-s/\tau_{f}^{\text{bAP}}}+e^{-s/\tau_{s}^{\text{bAP}}}\right), \end{array}$$

with *A*_bAP_ = 100 mV, τ^bAP^_*f, s*_ = 3, 25 ms, *I*_^bAP^*f, s*_ = 0.75, 0.25, and τ^EPSP^_*f, s*_ = 5, 50 ms. *A*_EPSP_ depends on the time constants such that the maximum of the EPSP is exactly 1 mV. The model assumes that the only source of calcium into the postsynapse are the NMDA receptors. Each presynaptic spike opens a fraction of *P*_0_ = 0.5 of all receptors still in the closed state, and in inter spike intervals the open receptors decay exponentially back to the closed state, however, with two components with different time constants. This translates to the following equations, where *NMDA*(*t*) is the fraction of open receptors at time *t*:

(24) $$\begin{array}{l} \;{\dot{N}}_{f}=-\frac{N_{f}}{\tau_{f}}+P_{0}I_{f}(1-\text{NMDA }(t-0))x_{\text{pre}}\\ \;{\dot{N}}_{s}=-\frac{N_{s}}{\tau_{s}}+P_{0}I_{s}(1-\text{NMDA }(t-0))x_{\text{pre}}\\ \text{NMDA}(t)=N_{f}(t)+N_{s}(t)\;. \end{array}$$

*I*_*f, s*_ = 0.5, 0.5 set the relative amplitudes of the fast and the slow component, which decay back with time constants τ_*f, s*_ = 50, 200 ms. Due to the voltage-dependent magnesium block of NMDA receptors, the resulting calcium current is a function of both the fraction of open receptors as well as the membrane potential:

(25) $$\begin{array}{l} I_{\text{NMDA}}(t)=G_{\text{NMDA}}\cdot \text{NMDA}(t)(V(t)-V_{r})B(V)\;,\text{ with}\\ \;B(V)={\left(1+0.28\exp\left(-0.062V\right)\right)}^{-1}\;, \end{array}$$

with *V* in mV, *V*_*r*_ = 130 mV and $G_{\text{NMDA}}=-0.02\frac{\mu M}{\text{ms}\cdot \text{mV}}$. The calcium concentration is a low pass filtered version of the current, with decay time constant τ_*Ca*_:

(26) $$\frac{d[Ca](t)}{dt}=I_{\text{NMDA}}(t)-\frac{\left[Ca](t\right)}{\tau_{Ca}}\;.$$

The central assumption in this model is now that synaptic change is completely ruled by the concentration of calcium in the postsynaptic spine. Low concentrations lead to LTD, high concentrations lead to LTP. Also, the learning rate is a monotonic function of calcium concentration:

(27) $$\begin{array}{l} \eta ([Ca])={\left(\frac{0.1s}{{\left[Ca\right]}^{3}+10^{-5}}+1s\right)}^{-1}\\ \Omega ([Ca])=0.25+\text{sig}([Ca]-\alpha_{2},\beta_{2})-0.25\text{sig}([Ca]-\alpha_{1},\beta_{1})\\ \text{sig}(x,\beta )=\exp(\beta x)/(1+\exp(\beta x)), \end{array}$$

where [*Ca*] is measured in μ*M* [in Equation 26 it is measured in *mM*]. The resulting weight change is finally:

(28) $$\dot{W}=\eta ([Ca])\left(\Omega ([Ca])-W(t)\right)$$

### STDP and mean weight change with oscillating firing rates

In the mean field case, we investigated the rate of weight change in spSTDP with periodically oscillating firing rates. The rate as a function of time is given by Equation (18) We solved Equation (15) with these *r*_*i*_ (*t*). After sufficient time (*t* » τ_*i*_), the transient is gone, and the solution for the traces is

(29) $$y_{i}=\tau_{i}\left[1+\frac{\varepsilon \cos\left(\omega t-\phi_{i}+\arctan(-\omega \tau_{i})\right)}{\sqrt{1+\omega^{2}\tau_{i}^{2}}}\right]\;,$$

where we replaced ω = 2π*f*_mod_ for convenience. We now assume stable conditions, and calculate the rate of weight change:

(30) $$\Delta w=\frac{1}{T}\int_{0}^{T}\left(qy_{\text{pre}}r_{\text{post}}-\frac{y_{\text{pre}}y_{\text{post}}}{\tau_{\text{post}}}\right)dt\;.$$

We replace $A_{i}=\varepsilon /\sqrt{1+\omega^{2}\tau_{i}^{2}}$ and ψ_*i*_ = arctan(−ωτ_*i*_) and get the solution:

(31) $$\begin{array}{l} \Delta w=\Delta w_{+}+\Delta w_{-}\\ \;=\tau_{\text{pre}}(q-1)+\frac{A_{\text{pre}}}{2}(\varepsilon \cos(\psi_{\text{pre}}+\Delta \varphi )-A_{\text{post}}\cos(\psi_{\text{pre}}+\Delta \varphi -\psi_{\text{post}})). \end{array}$$

### Derivation of *f*_max_

To gain insight into the reasons for theta susceptibility, we investigate spSTDP in the balanced case (*q* = 1). We are interested in the oscillation frequency at which the difference between potentiation and depression depending on phase difference becomes maximal. Instead of computing the derivative of Equation (31) with respect to ω, we explicitly use the functional form of *y*_pre_ and *ẏ*_post_ (see Equation 29):

(32) $$\begin{array}{l} \;y_{\text{pre}}=\tau_{\text{pre}}\left[1+A_{\text{pre}}\cos\left(\omega t+\psi_{\text{pre}}\right)\right]\\ {\dot{y}}_{\text{post}}=\omega \tau_{\text{post}}A_{\text{post}}\cos\left(\omega t+\psi_{\text{post}}-\Delta \varphi -\pi /2\right). \end{array}$$

*y*_pre_ is bounded between 1 + ε and 1 − ε, and because of 0 < ε < 1 it is strictly positive. The weight change is the integral of the product of both functions, therefore *y*_pre_ acts as a weighting function for *ẏ*_post_. As a consequence, potentiation is maximal if the maxima of both functions coincide, i.e., the phases have to be identical. This is true for Δφ = ψ_post_ − ψ_pre_ − π/2 (Shifted by π for depression). At this phase shift, the rate of weight change becomes

(33) $$\Delta w=\frac{1}{T}\int_{0}^{T}y_{\text{pre}}{\dot{y}}_{\text{post}}dt=\frac{1}{2}\frac{\varepsilon^{2}\tau_{\text{pre}}\tau_{\text{post}}}{\sqrt{1+\omega^{2}\tau_{\text{pre}}^{2}}\sqrt{1+\omega^{2}\tau_{\text{post}}^{2}}}.$$

We calculate the derivative with respect to ω:

(34) $$\frac{2}{\varepsilon^{2}\tau_{\text{pre}}\tau_{\text{post}}}\frac{d\Delta w}{d\omega }=\frac{1}{\sqrt{1+\omega^{2}\tau_{\text{pre}}^{2}}\sqrt{1+\omega^{2}\tau_{\text{post}}^{2}}}\cdot \left(1-\frac{\tau_{\text{pre}}^{2}\omega^{2}}{1+\tau_{\text{pre}}^{2}\omega^{2}}-\frac{\tau_{\text{post}}^{2}\omega^{2}}{1+\tau_{\text{post}}^{2}\omega^{2}}\right)$$

To find the maximum ω_max_, we set *d*Δ*w*/*d*ω = 0 and solve for ω:

(35) $$f_{\text{max}}=\frac{\omega_{\text{max}}}{2\pi }=\frac{1}{2\pi \sqrt{\tau_{\text{pre}}\tau_{\text{post}}}}\;.$$

### Limits of parameter values in the fitting process

The fitting process of the CD model and the Triplet model to the data was a brute force search in parameter space. For that, we defined a volume of space wherein the search was conducted. The bounds for that space are given in Table 1 for the CD model and Table 2 for the Triplet model. The range of possible values for ϑ_*q*_ is given by [0, 0.2], with one additional value <0, whose magnitude does not matter. Except in two cases the parameters always sat well within this space. In the case of the data in HC (Wang et al., 2005), the presynaptic adaptation time constant τ^rec^_pre_ should be long, and the optimum lies at even longer times than given in Table 3. However, the influence of this parameter on the error is very small, and changes in τ^rec^_pre_ do not change the other parameters much. Therefore we decided to set it to 3 s. In the Triplet model, in the fit to the data in SC23, the parameters τ_*x*_ and *A*^+^_3_ lie outside the bounds. When the first fit showed that those parameters hit the bounds, we decided to redefine the parameter space for this data set, in order to find a good minimum.
